# Application of Capillary and Free-Flow Zone Electrophoresis for Analysis and Purification of Antimicrobial β-Alanyl-Tyrosine from Hemolymph of Fleshfly *Neobellieria bullata* †

**DOI:** 10.3390/molecules26185636

**Published:** 2021-09-16

**Authors:** Veronika Šolínová, Petra Sázelová, Alice Mášová, Jiří Jiráček, Václav Kašička

**Affiliations:** Institute of Organic Chemistry and Biochemistry of the Czech Academy of Sciences, Flemingovo n. 542/2, 166 10 Prague 6, Czech Republic; veronika.solinova@uochb.cas.cz (V.Š.); petra.sazelova@uochb.cas.cz (P.S.); alice.masova@gmail.com (A.M.); jiri.jiracek@uochb.cas.cz (J.J.)

**Keywords:** antimicrobial peptides, beta-alanyl-tyrosine, capillary zone electrophoresis, free-flow zone electrophoresis, peptide analysis, peptide purification

## Abstract

The problem of a growing resistance of bacteria and other microorganisms to conventional antibiotics gave rise to a search for new potent antimicrobial agents. Insect antimicrobial peptides (AMPs) seem to be promising novel potential anti-infective therapeutics. The dipeptide β-alanyl-tyrosine (β-Ala-Tyr) is one of the endogenous insect toxins exhibiting antibacterial activity against both Gram-negative and Gram-positive bacteria. Prior to testing its other antimicrobial activities, it has to be prepared in a pure form. In this study, we have developed a capillary zone electrophoresis (CZE) method for analysis of β-Ala-Tyr isolated from the extract of the hemolymph of larvae of the fleshfly *Neobellieria bullata* by reversed-phase high-performance liquid chromatography (RP-HPLC). Based on our previously described correlation between CZE and free-flow zone electrophoresis (FFZE), analytical CZE separation of β-Ala-Tyr and its admixtures have been converted into preparative purification of β-Ala-Tyr by FFZE with preparative capacity of 45.5 mg per hour. The high purity degree of the β-Ala-Tyr obtained by FFZE fractionation was confirmed by its subsequent CZE analysis.

## 1. Introduction

Excessive use of antibiotics in human and veterinary medicine and agriculture farming in the last several decades almost all over the world caused the selection and spread of antibiotic-resistant microorganisms. These microbes are considered as one of the major threats of public health [1,2]. Hence, there is an urgent need for looking for novel non-conventional antimicrobial compounds, which should become new antibiotic drugs in the next years [3,4,5]. Antimicrobial peptides (AMPs) are considered as a promising group of such new anti-infective agents with an in principle different mechanism of action and faster killing process than the current conventional antibiotics [3,6,7].

AMPs are a part of the innate defense system of all living organisms, and they usually comprise from 10 to 50 amino acid residues. A large variety of AMPs has been identified across species with over 20,000 experimentally validated peptides reported so far [8]. Among them, the insect AMPs are especially strongly investigated as promising candidates for new antimicrobials because insects represent the largest and most biodiverse class of organisms in the animal kingdom [5,9]. Their humoral immune response to microbial infection includes synthesis of AMPs and their release into the insect hemolymph.

The mechanism of antimicrobial action is largely based on electrostatic interaction of cationic peptides with anionic phospholipids of the microbial cell membrane leading to disintegration/perturbation of the latter, and resulting in cell death [3,4,7]. AMPs have also been found to exert activities such as neutralization of the pro-inflammatory agent (lipopolysaccharide), immune cell chemo-attraction, and cell proliferation. This has led to the hope of potential development of AMPs as novel anti-infective agents with additional beneficial properties [3,4,5,9].

**Figure 1:**
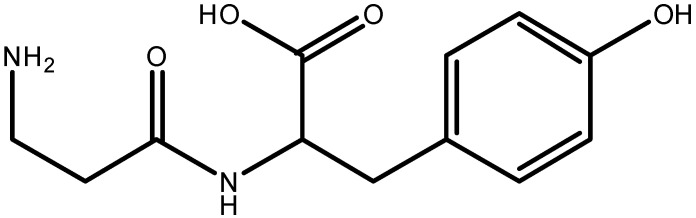
Molecular structure of the dipeptide β-alanyl-tyrosine.

Dipeptide β-alanyl-tyrosine (β-Ala-Tyr), see Figure 1, is one of the endogenous insect toxins called paralysins. They are known to cause, upon injection, paralysis or even death of the adults of the same and other insect species. β-Ala-Tyr was first reported in 1969 as “sarcophagine” present in the fleshfly *Neobellieria (Sarcophaga) bullata* [10]. It was found to be synthesized in fat body and to be accumulated in the larval hemolymph up to the formation of the white puparium. At the moment of pupariation, the concentration of β-Ala-Tyr dramatically decreases due to its degradation into the constituent amino acids β-Ala and Tyr that are subsequently involved in melanisation and sclerotisation of the cuticle [11].

β-Ala-Tyr belongs to the group of peptide antimicrobials, and it shows an antibacterial activity against both Gram negative and Gram positive bacteria and antifungal activity as well [12,13]. In the current project, β-Ala-Tyr was found in one of the fractions of the reversed-phase high-performance liquid chromatography (RP-HPLC) separation of the solid phase extract from the hemolymph of *Neobellieria bullata* raising an immunity response against microbial infection. The compounds contained in these fractions should be tested for their antimicrobial activity against *Escherichia coli* and *Staphylococcus aureus.* Prior to their application in biological tests, these compounds should be separated and isolated in a pure form.

Peptides are traditionally purified by liquid chromatographic methods, especially RP-HPLC. However, due to weak retention of short hydrophilic peptides on the reversed phases, the structurally similar peptides often co-elute in the same fraction. For that reason, another method, based on a different separation principle, should be used for purity control of peptides separated by RP-HPLC. This criterion is well met by capillary zone electrophoresis (CZE), which separates the compounds on the basis of their different electrophoretic mobilities, i.e., according to their charge/hydrodynamic radius (molecular mass) ratio. CZE is appreciated as a powerful analytical technique featuring high separation efficiency (hundreds of thousands to millions of theoretical plates), high substance amount/mass sensitivity (picomole to attomole range in nanoliter to picoliter injected volumes), and short time of analysis (typically 10–20 min). CZE represents a suitable complement and/or alternative to different modes of HPLC and possesses high application potential for analysis and characterization of amino acids [14], peptides [15], proteins [16], and other (bio)molecules [17,18].

However, application of CZE for preparative separation of peptides and other (bio)molecules is limited. Partially, due to more complicated adaptation of analytical CZE setups to preparative ones than in the case of HPLC, but mainly because of the inherently low preparative capacity of narrow bore capillaries with inner diameter ≤ 100 μm. The former problem (caused by dipping of both capillary ends into the solution in electrode vessels at high separation voltage during the whole CZE analysis) can be solved by short interrupting of electric field and using autosamplers as fraction collectors [19,20]. However, only small enhancement of preparative capacity can be gained by increasing the capillary inner diameter because of worse Joule heat removal and reduced separation efficiency in wider bore capillaries. The principal solution of the problem of low preparative capacity of CZE separations is to perform the electrophoretic separations in the free-flow arrangement, i.e., to convert analytical CZE separation into preparative free-flow zone electrophoresis (FFZE) fractionation performed in the free-flow chamber [21].

**Figure 2:**
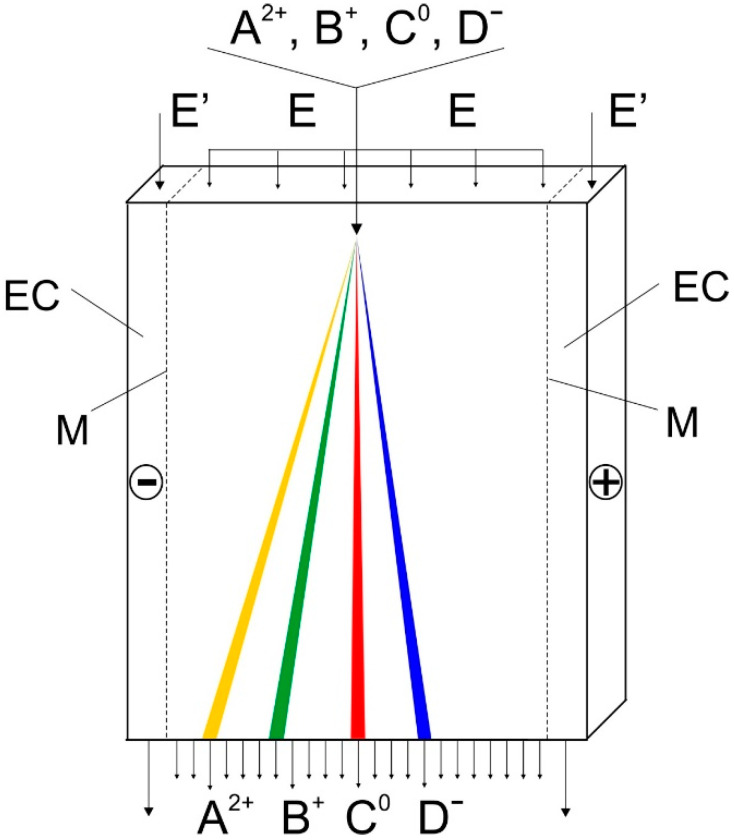
Scheme of the flow-through electrophoretic chamber and principle of the free-flow zone electrophoresis (FFZE). A^2+^, B^+^, C^0^, and D^−^, sample components continuously introduced to the injection point of the chamber as a narrow zone and collected at the outlet side of the chamber; E, carrier background electrolyte (BGE) continuously introduced by a laminar flow into the chamber and collected at its outlet side; E’, BGE circulating in the electrode compartments (EC); M, ion-exchange membranes between the separation chamber and the electrode compartments.

In the FFZE, an electric field is oriented perpendicularly to the laminar flow of the carrier background electrolyte (BGE) in the narrow gap between two parallel glass plates, see Figure 2. Sample solution is continuously injected into the carrier BGE as a narrow zone. The charged sample components are deflected from the straight direction of laminar flow by the d.c. electric field at different angles directly proportional to their electrophoretic mobilities (the higher mobility, the larger deflection angle). At the outlet side of the chamber, the carrier BGE and the separated sample components are continuously collected in the fraction collector.

FFZE has been applied for preparative separation and purification of many types of charged (bio)molecules and (bio)particles [22], e.g., amino acids and peptides [23,24], proteins [25,26,27], cells [28], and cell organelles [29]. In some cases, FFZE has been employed as the first dimension in the multidimensional separation of complex mixtures of peptides and proteins in proteomic and peptidomic studies, e.g., in a combination with RP-HPLC-MS/MS [30,31] and MS [32,33]. Microchip formats of FFZE and other electrophoretic methods, e.g., free-flow isotachophoresis (FFITP) [34] and free-flow isoelectric focusing (FFIEF) [27] have been used for continuous microscale separation of analytes prior to their UV-absorption, fluorescence, or MS detection, for reviews see [35,36].

Since CZE and FFZE are based on the same separation principle, zone electrophoresis (ZE), and both are performed in a free (carrierless) medium using the same composition of BGE, there is a direct correlation between these two methods [37,38,39]. Based on this correlation, the analytical CZE separation can be converted to preparative FFZE fractionation [21,37,38,39,40].

The aim of this work was (i) to apply CZE for determination of purity of β-Ala-Tyr isolated by RP-HPLC separation of the extract from the hemolymph of larvae of the fleshfly *Neobellieria bullata*, (ii) to convert the analytical CZE separation β-Ala-Tyr and its admixtures into preparative FFZE fractionation, (iii) to utilize the developed FFZE fractionation for purification of β-Ala-Tyr with the preparative capacity of tens of milligram per hour for the purpose of testing its antimicrobial activity, and (iv) to check the purity of β-Ala-Tyr obtained in the FFZE fractions by CZE.

## 2. Experimental

### 2.1. Chemicals and Materials

All chemicals used were of analytical grade. Acetic acid, sodium hydroxide, and phenol were obtained from Lachema (Brno, Czech Republic). Acetonitrile (ACN) and trifluoroacetic acid (TFA) were purchased from Sigma-Aldrich Ltd. (Prague, Czech Republic). Diglycine and triglycine were supplied by Reanal (Budapest, Hungary). The background electrolyte (BGE) was prepared from the deionized Milli-Q water and was filtered through a 0.45 μm syringe filter (Millipore, Bedford, MA, USA) before use.

### 2.2. Sample Preparation

The third-instar larvae of the fleshfly *Neobellieria bullata* were injected with bacterial suspension of *Escherichia coli* or *Staphylococcus aureus,* and then, after 24 h, the hemolymph was collected and preseparated by solid phase extraction into hydrophilic and hydrophobic fractions using Chromabond^®^C-18 cartridges (Macherey-Nagel, Düren, Germany). Hydrophilic fractions were eluted by 0.1% (*v*/*v*) TFA and hydrophobic fractions by 80% (*v*/*v*) aqueous ACN. These fractions were separated by RP-HPLC using the Waters 600 HPLC system and the C-18 columns Vydac 218TP54 (250 × 4 mm, 5 μm, or 250 × 10 mm, 5 μm). Sample components were eluted by gradient elution with increasing concentration of ACN in water in the presence of 0.1% (*v*/*v*) TFA. Isolated fractions were characterized by UV-Vis spectrophotometry, amino acid analysis, ESI-MS, MALDI-MS, SDS-PAGE, tryptic digest, and *N*-terminal sequencing.

### 2.3. Instrumentation

#### 2.3.1. Capillary Zone Electrophoresis

The CZE analyses were carried out in commercial P/ACE MDQ System (Beckman-Coulter, Fullerton, CA, USA), data acquisition and evaluation were performed using the software P/ACE System MDQ, version Karat 7, supplied by Beckman, and the Chromatography and Electrophoresis Station Clarity (DataApex, Prague, Czech Republic). The apparatus was equipped with the bare fused silica capillary with outer polyimide coating, total/effective length 393/290 mm, id/od 50/375 μm (Polymicro Technologies, Phoenix, AZ, USA). The BGE was composed of 500 mM acetic acid, pH 2.50, separation voltage was 20.0 kV, and electric current was 11.8 μA. Sample solutions were injected hydrodynamically (pressure 6.9 mbar × 5–10 s). The analytes were detected by UV-Vis spectrophotometric photodiode array detector set at 195 and 206 nm. The temperature of liquid coolant of the capillary was 20 °C.

The new fused silica capillary was gradually flushed with water, 1 M NaOH, water, and BGE, each wash for 10 min by pressure of 1–2 bar. Finally, the capillary was conditioned by a 20 min application of the separation voltage to equilibrate the inner surface with BGE and to stabilize the electroosmotic flow (EOF). Between runs under the same conditions, the capillary was rinsed with the BGE for 2 min by pressure of 1 bar.

#### 2.3.2. Free-Flow Zone Electrophoresis

FFZE experiments were performed in an adapted home-made apparatus [41]. The core of this device is a flow-through electrophoretic chamber consisting of two parallel glass plates (500 × 500 × 4 mm) with a 0.5 mm gap between them. The carrier BGE (500 mM acetic acid, pH 2.50) was introduced through the six inlets by six-piston pump with a flow-through (residence) time of 31 min. The sample was continuously injected by a peristaltic pump with a flow-rate of 1.6 mL/h. The effective length of the separation trajectory (from the sample inlet position to the chamber outlet) was 440 mm. Both outer sides of the glass plates of the chamber were cooled by the fast-flowing air to the temperature of −1 °C, the temperature inside the chamber was +4 °C. Separations were performed in the constant voltage regimen (3.0 kV, 122–125 mA). At the outlet side of the chamber, the carrier BGE and the sample components were collected in 48 fractions and periodically sucked-off into the large volume fraction collector. The collected fractions were evaluated by off-line UV-absorption measurement at 220 and 280 nm and/or by CZE analysis.

## 3. Theoretical Background

### Correlation between CZE and FFZE

**Figure 3:**
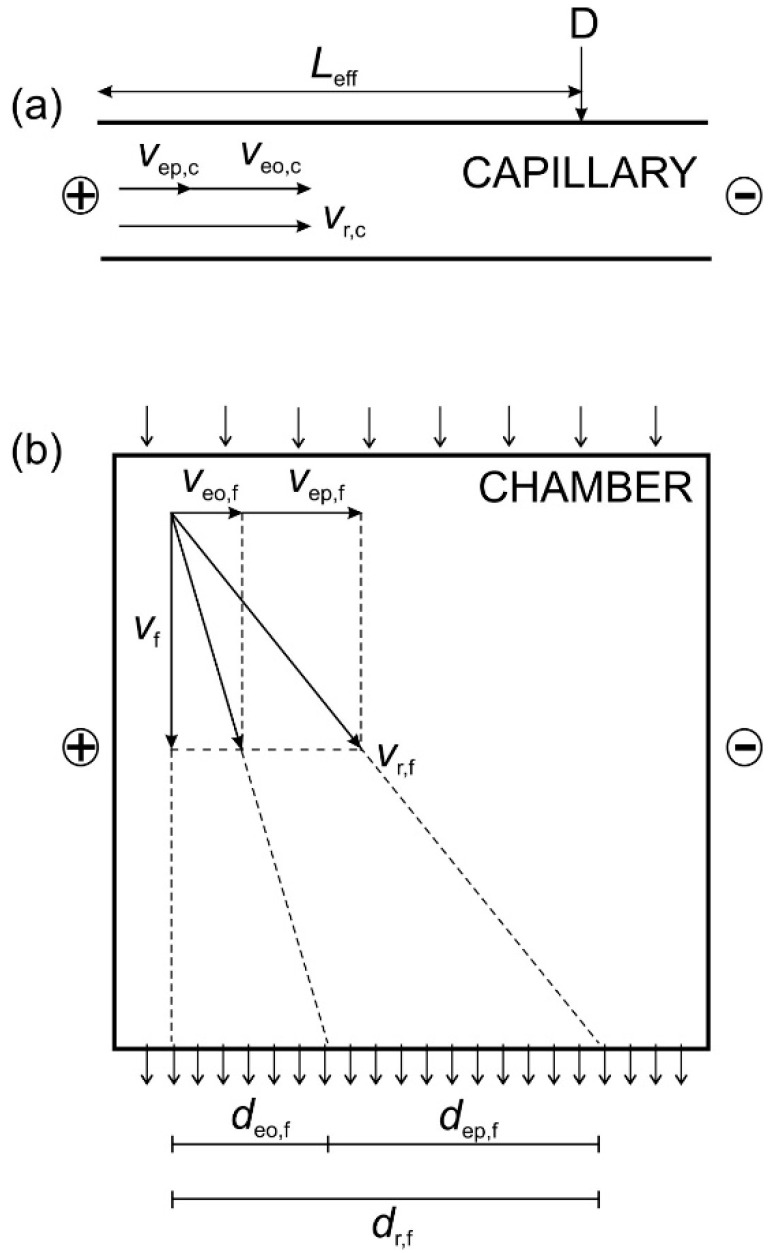
Superposition of the migration velocities in CZE (**a**) and FFZE (**b**); *L*_eff_, effective length of the capillary; D, detection position; *v*_ep,c_, electrophoretic velocity in CZE; *v*_eo,c_, electroosmotic velocity in CZE; *v*_r,c_, resulting migration velocity in CZE; *v*_ep,f_, electrophoretic velocity in FFZE; *v*_eo,f_, electroosmotic velocity in FFZE; *v*_f_, hydrodynamic flow velocity in FFZE; *v*_r,f_, resulting migration velocity in FFZE; *d*_eo,f_, electroosmotically migrated distance in FFZE; *d*_ep,f_, electrophoretically migrated distance in FFZE; *d*_r,f_, resulting migration distance in FFZE.

The detailed theoretical background of the correlation between CZE and FFZE for the conversion of analytical CZE to preparative FFZE was described elsewhere [38,39]. Here, only the main results will be presented. The analogy between CZE and FFZE, in particular, the vector superposition of the electrophoretic, electroosmotic and hydrodynamic migration velocities and of the migration distances in the capillary and in the flow-through electrophoretic chamber are shown in Figure 3.

The resulting migration velocity of the charged compound in the d.c. electric field in the capillary, *v*_r,c_, is equal to the sum of the electrophoretic velocity, *v*_ep,c_, and the electroosmotic velocity *v*_eo,c,_ respectively, see Figure 3a. For the electrophoretic velocity of the charged component in the capillary, *v*_ep,c_, the following relation was derived [39]:

(1) $$v_{\mathrm{ep},c}=\frac{L_{\mathrm{eff}}\left(t_{\mathrm{eo},c}-t_{r,c}\right)}{t_{\mathrm{eo},c}t_{r,c}}$$

where *L*_eff_ is the effective length of the capillary (from the injection end to the detector), *t*_r,c_ is the resulting migration time of the analyte in CZE, and *t*_eo,c_, is the migration time of the electroneutral EOF marker in CZE, respectively.

The resulting migration distance of a charged compound in the free-flow chamber, *d*_r,f_, is equal to the sum of the electrophoretically migrated distance, *d*_ep,f_, and the electroosmotically migrated distance, *d*_eo,f_, respectively, see Figure 3b. From this graph it follows that the electrophoretic velocity of the charged component in the chamber, *v*_ep,f_, is given by the following equation:

(2) $$v_{\mathrm{ep},f}=\frac{d_{r,f}-d_{\mathrm{eo},f}}{t_{f}}$$

where *t*_f_ is the mean flow-through time (also called residence time) of the carrier BGE in the chamber.

From the Equations (1) and (2), the ratio of the electrophoretic velocities in the chamber and in the capillary, *r*_ep_, can be expressed:

(3) $$r_{\mathrm{ep}}=\frac{v_{\mathrm{ep},f}}{v_{\mathrm{ep},c}}=\frac{t_{r,c}t_{\mathrm{eo},c}\left(d_{r,f}-d_{\mathrm{eo},f}\right)}{L_{\mathrm{eff}}t_{f}\left(t_{\mathrm{eo},c}-t_{r,c}\right)}$$

If adsorption of the sample components to the walls of the separation compartments (both fused silica capillary and glass chamber) can be neglected or is the same, it is reasonable to assume that the ratio *r*_ep_ is approximately constant for different charged components separated by CZE and FFZE in the same BGE. In other words, if the electrophoretic velocity of the standard component S in FFZE, *v*_ep,f,S_, is *r*_ep_-times higher than the electrophoretic velocity of this component in CZE, *v*_ep,c,S_, then, the electrophoretic velocity of peptide P in FFZE, *v*_ep,f,P_, analyzed under the same conditions as the standard component S, will be also *r*_ep_-times higher in FFZE than the electrophoretic velocity of peptide P in CZE, *v*_ep,c,P_, i.e.,:

(4) $$r_{\mathrm{ep}}=\frac{v_{\mathrm{ep},f,S}}{v_{\mathrm{ep},c,S}}=\frac{v_{\mathrm{ep},f,P}}{v_{\mathrm{ep},c,P}}$$

Consequently, the ratio *r*_ep_ (determined for standard component S) can be used to predict the electrophoretic velocities of sample components (peptide P and its admixtures) in FFZE, *v*_ep,f,P_, if their electrophoretic velocities in CZE, *v*_ep,c,P_, were determined under the same conditions as the electrophoretic velocity of the standard S.

(5) $$v_{\mathrm{ep},f,P}=r_{\mathrm{ep}}\times v_{\mathrm{ep},c,P}$$

The above equations represent basis for conversion of analytical CZE separations into preparative FFZE fractionations. When the ratio *r*_ep_ is once measured, it can be used to predict the migration distances of peptide P and its admixtures in the chamber.

## 4. Results and Discussion

### 4.1. CZE Analysis of β-Ala-Tyr Isolated from the Hemolymph of N. bullata by RP-HPLC

**Figure 4:**
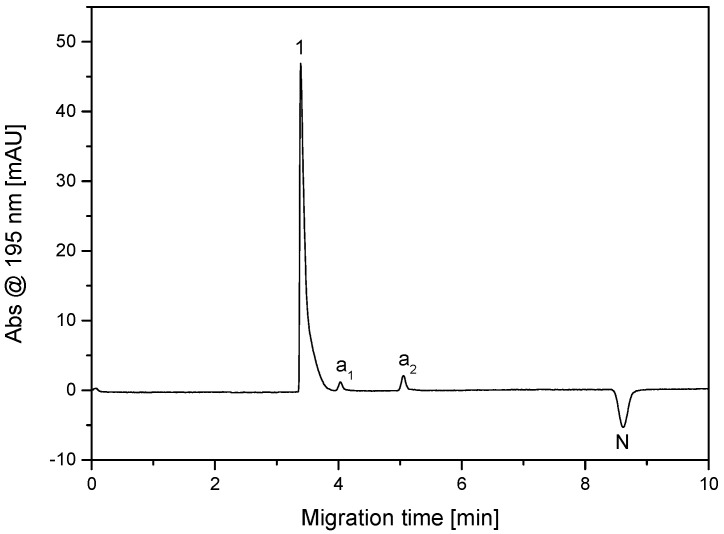
CZE analysis of dipeptide β-Ala-Tyr obtained by HPLC separation of an extract from the hemolymph of the larvae of *Neobellieria bullata*. 1, β-Ala-Tyr; a_1_, a_2_, non-identified admixtures; N, negative peak of water zone (with migration time identical as that of the neutral EOF marker (phenol)). BGE: 500 mM AcOH, pH 2.50; capillary: bare fused silica, total/effective length 393/290 mm, id/od 50/375 μm; separation voltage: 20.0 kV; current 11.8 μA. Sample concentration 0.5 mg/mL. For other experimental conditions, see Section 2.3.1.

The dipeptide β-Ala-Tyr was isolated as a main component from one of the fractions obtained by the RP-HPLC separation of the solid phase extract of hemolymph of the larvae of *Neobellieria bullata* using the above procedure, see Section 2.2. Based on our previous experience with CZE analysis of antimicrobial and other peptides [15,42,43], the amphoteric dipeptide β-Ala-Tyr was analyzed as a cation in acidic BGE composed of 500 mM AcOH, pH 2.50, under the conditions specified in the Section 2.3. Record of this CZE analysis is shown in Figure 4. In addition to the main component of this fraction, β-Ala-Tyr (peak 1), two admixtures, peaks a_1_ and a_2_, can be observed on the electropherogram. The negative peak N corresponds to the non-absorbing water zone (the sample was dissolved in water), the migration time of which was identical with the migration time of the electroneutral EOF marker (phenol) in the CZE separation of standard components (diglycine, triglycine, and phenol). CZE and FFZE separations of the standard components were used for determination of the ratio of electrophoretic and electroosmotic velocities in CZE and FFZE. The purity degree of β-Ala-Tyr was evaluated as a ratio of the corrected (migration time normalized) peak area of this dipeptide and the sum of the corrected (migration times normalized) peak areas of all components found in this sample (fraction) by CZE analysis [44]. The purity degree of the β-Ala-Tyr reached the value of 94.8%. For antimicrobial tests, it was necessary to purify β-Ala-Tyr, in order to prevent influence of these two admixtures on its antimicrobial activity. Due to the presence of the charged admixtures a_1_ and a_2_ in the β-Ala-Tyr RP-HPLC fraction, it was decided to use FFZE for final preparative purification of β-Ala-Tyr.

### 4.2. Conversion of Analytical CZE Separation to Preparative FFZE Fractionation

Based on the above-described correlation between CZE and FFZE, the analytical CZE separation was converted into preparative FFZE fractionation. It consisted of the following steps:

1. From the parameters of CZE analysis of β-Ala-Tyr (further in this section indicated as peptide P), shown in Figure 4 (the effective capillary length, *L*_eff_; the resulting migration time of this peptide, *t*_r_; and the migration time of the electroneutral EOF marker, *t*_eo_), the electrophoretic velocity of this peptide in the capillary, *v*_ep,c,P_, was calculated using Equation (1).
2. From the parameters of the CZE separation of standard components (diglycine and triglycine, further in this section indicated as “general standard component S”) and neutral EOF marker N (phenol) carried out under the same conditions as the CZE analysis of the above peptide P (see Figure S1 in the Supporting Materials), the electrophoretic velocity of the standard component S, *v*_ep,c,S_, was calculated using Equation (1).
3. Standard components (diglycine and triglycine) as representatives of the general standard component S, and the electroneutral EOF marker N (phenol) were separated by FFZE using the same BGE as in CZE (see Figure S2 in the Supplementary Materials). From this experiment, the resulting migrated distances of standard S, *d*_r,f,S_, and the EOF migrated distance, *d*_eo,f_ (equal to migration distance of phenol), in the FFZE were obtained. They were used for calculation of the electrophoretic velocity of standard component S in chamber, *v*_ep,f,S_, using Equation (2).
4. From the results obtained in steps 2 and 3, the ratio of the electrophoretic velocities of the standard component S in the chamber and in the capillary, *r*_ep,S_, was determined:

(6) $$r_{\mathrm{ep},S}=\frac{v_{\mathrm{ep},f,S}}{v_{\mathrm{ep},c,S}}$$

5 Based on the results obtained in steps 1 and 4, and taking into account that the ratio of electrophoretic velocity in the chamber and in the capillary is identical for all charged components, the electrophoretic velocity of peptide P in FFZE, *v*_ep,f,P_, could be calculated using the ratio *r*_ep,S_ determined for standard component S (*v*_ep,f,P_ = *r*_ep,S_ × *v*_ep,c,P_). The predicted resulting migration distance of peptide P in the chamber, *d*_r,f,pred,P_, was obtained as a sum of the EOF moved distance in the chamber, *d*_eo,f_, and the electrophoretically moved distance, *d*_ep,f,P_, (*d*_ep,f,P_ = *v*_ep,f,P_ × *t*_f,_):

*d*_r,f,pred,P_ = *d*_eo,f_ + *r*_ep,S_ × *v*_ep,c,P_ × *t*_f_ (7)

6 The predicted resulting migration distances in FFZE can be calculated also for the other charged components of the peptide sample, e.g., admixtures a_1_ and a_2_ in this particular case, and their separability in FFZE can be estimated.

The particular values of the measured resulting migration times and calculated electrophoretic and electroosmotic velocities of the standard components S_1_ and S_2_ (diglycine and triglycine) in CZE and their resulting migration distances in FFZE, and the ratio of their electrophoretic and electroosmotic velocities in the FFZE and CZE are summarized in Table 1. The measured resulting migration times and calculated electrophoretic and electroosmotic velocities and effective mobilities of the dipeptide β-Ala-Tyr and its admixtures (a_1_ and a_2_) in CZE are presented in Table 2, column CZE, and the predicted migration distances of the dipeptide β-Ala-Tyr and its admixtures a_1_ and a_2_, are shown in Table 2, in the section CZE and in the section FFZE, the column *d*_r,pre_, respectively.

According to the predicted migration distances of the sample components, see the column *d*_r,pre_, in Table 2, the conditions of FFZE should be sufficient for separation β-Ala-Tyr and its admixtures also in the preparative scale with high concentration of the sample introduced in the flow-through chamber.

According to the predicted migration distances of the sample components, the separation conditions of FFZE, namely separation voltage and/or flow-through time, are optimized. If the differences in the predicted distances are too small and the separation of sample components is not sufficient, then, the voltage and/or flow-through time should be increased. If the predicted distances for the sample components are too long and there is a danger that the fastest component could reach the close vicinity of the ion-exchange membrane separating the separation chamber from the electrode vessel, where this component can be damaged or lost because of concentration, pH and conductivity non-homogeneities occurring in this region, then the voltage and/or flow-through time must be decreased.

In this way, the correlation of CZE and FFZE allows to develop the suitable separation conditions in more economical and faster microscale by CZE than by FFZE and only, then the optimized conditions can be converted into the preparative FFZE fractionation.

All these characteristics, including effective electrophoretic mobilities of the involved species corrected to 25 °C, are presented in Table 2. The predicted migration distances (20–119 mm) and the differences in migration distances between sample individual components at the outlet side of the chamber indicated that a sufficient separation could be achieved in the same FFZE procedure, which was used for separation of the standard components.

### 4.3. Purification of Isolated β-Ala-Tyr by FFZE

**Figure 5:**
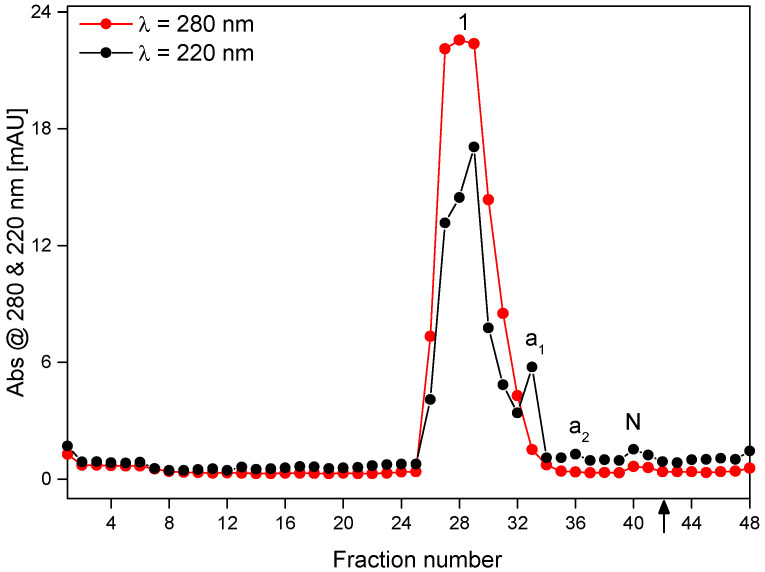
FFZE separation of dipeptide β-Ala-Tyr obtained by HPLC separation of an extract from the hemolymph of the larvae of *Neobellieria bullata*. 1, β-Ala-Tyr; a_1_, a_2_, non-identified admixtures; N, noncharged sample component. Carrier BGE: 500 mM AcOH, pH 2.50; separation voltage 3.0 kV; current 122–125 mA; sample flow rate 1.6 mL/h; flow-through time 31 min; sample concentration 30 mg/mL, total sample amount 63.8 mg. The arrow at fraction no. 42 indicates the position, at which the sample is continuously introduced at the inlet side of the flow-through (free-flow) chamber. For other experimental conditions, see Section 2.3.2.

For purification of the β-Ala-Tyr sample obtained by RP-HPLC, the same FFZE conditions as those used for separation of the standard components have been applied (mean flow-through time of 31 min, separation voltage of 3.0 kV, sample flow-rate of 1.6 mL/h, 500 mM AcOH as BGE, see the Section 2.3.2). These parameters were derived from the experimental conditions of CZE and the obtained CZE data (electrophoretic velocities and electroosmotic velocities). The lyophilizate of the isolated HPLC fraction containing β-Ala-Tyr (63.8 mg) was dissolved in the BGE (0.5 M AcOH) at concentration of 30 mg·mL^−1^ and applied to FFZE fractionation. The record of FFZE separation of components present in this HPLC fraction using the off-line UV-absorption detection at 220 nm and 280 nm, respectively, is depicted in Figure 5. Apparently, the FFZE separation is less efficient than CZE but in general, the separation profiles of CZE (Figure 4) and FFZE (Figure 5) are similar, the main peak 1 corresponding to β-Ala-Tyr and two smaller peaks corresponding to the admixtures a_1_ and a_2_ can be seen both in the CZE (Figure 4) and FFZE (Figure 5) separations. The differences in relative peaks heights are caused by the different detection wavelength in CZE and FFZE. Lower separation efficiency and resolution power of FFZE than CZE can be explained by less suitable conditions of much lower sample concentrations and higher electric field strength and temperature in CZE than in FFZE and the different material and dimensions of the separation compartment in both methods.

The real migration distances of β-Ala-Tyr and its admixtures a_1_ and a_2_ and the neutral component N determined from the record of FFZE experiment, *d*_r,exp_ (120, 90, 60, and 20 mm) are in good agreement with their predicted distances *d*_r,pre_ (119, 93, 65, and 20 mm), see Table 2.

From the above experimental data, the preparative capacity of the FFZE separation for the pure β-Ala-Tyr was calculated as a product of the sample concentration of 30 mg/mL, the relative content of β-Ala-Tyr in the original sample of 0.948 (taken as equal to the above purity degree of this peptide in the original sample—94.8%), and the sample flow-rate of 1.6 mL/h. The preparative capacity = 30 mg/mL × 0.948 × 1.6 mL/h = 45.5 mg/h.

### 4.4. CZE Analysis of FFZE Fractions Containing β-Ala-Tyr and Its Admixtures

**Figure 6:**
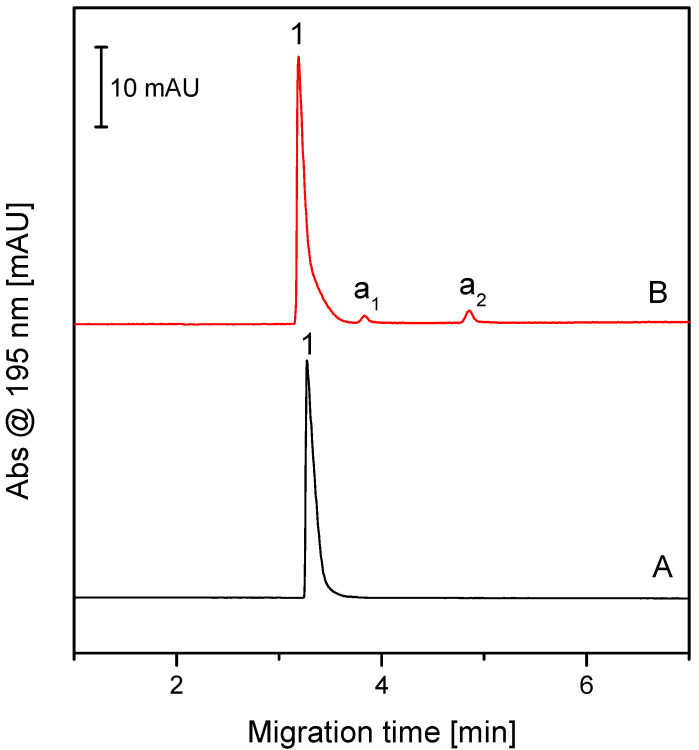
Comparison of CZE analysis of the dipeptide β-Ala-Tyr after (A) and before (B) its purification by FFZE. For the experimental conditions, see Figure 4 and Section 2.3.1.

The FFZE fractions containing UV-absorbing components were analyzed by CZE under the same conditions as the original β-Ala-Tyr sample. The representative result of these analyses, particularly the CZE electropherogram of the joint fractions 27–29, is presented in Figure 6, record 6A. Its comparison with the record 6B (CZE analysis of the original β-Ala-Tyr sample) shows that the joint fractions 27–29 (belonging to the top of the peak of the FFZE separation in Figure 5) contain pure β-Ala-Tyr, free of admixtures a_1_ and a_2_.

Hence, CZE analyses of FFZE fractions have shown that FFZE fractionation enabled to isolate β-Ala-Tyr in the pure form, free of admixtures a_1_ and a_2_.

## 5. Conclusions

Combined application of CZE and FFZE proved to be a useful approach for analysis and purification of the antimicrobial dipeptide β-Ala-Tyr isolated from the hemolymph of *Neobellieria bullata* by RP-HPLC. First, CZE was applied for analysis of this peptide and for the development of suitable experimental conditions for separation of admixtures of this peptide in a microscale. Then, based on the correlation between CZE and FFZE, the optimized CZE experimental conditions were converted to the FFZE fractionation. Thus, the development costs and time for peptide purification were reduced. FFZE enabled purification of β-Ala-Tyr with preparative capacity of 45.5 mg per hour. The advantage of the FFZE is that it runs continuously in a carrierless medium under mild conditions, at which the biological activity of the separated peptides is conserved, and the loss of peptide material is minimized. Finally, the high purity of β-Ala-Tyr prepared by the FFZE fractionation was confirmed by its CZE analysis.

## Figures and Tables

**Table 1:** Migration times, migration distances, electrophoretic and electroosmotic velocities of standard components, S_1_, diglycine, and S_2_, triglycine, and the electroosmotic flow marker, phenol, separated by CZE and FFZE.

Standard Component	CZE			FFZE			FFZE/CZE
	*t*_r,c_ [s]	*v*_ep,c_ [mm.s^−1^]	*v*_eo,c_ [mm.s^−1^]	*d*_r,f_ [mm]	*v*_ep,f_ [mm.s^−1^]	*d*_eo,f_ [mm]	*r*_ep_
S_1_, diglycine	162.5	1.219	0.565	160	0.0855	20	0.0701
S_2_, triglycine	179.0	1.055	0.565	140	0.0733	20	0.0695
N, phenol	513.2 *	0	0.565	20	0	20	-

*t*_r,c_, the migration time in CZE; *v*_ep,c_, the electrophoretic velocity in CZE; *v*_eo,c_, the electroosmotic velocity in CZE; *v*_ep,f_, the electrophoretic velocity in FFZE; *v*_eo,f_, the electroosmotic velocity in FFZE; *d*_r,f_, the resulting migration distance in FFZE; *r*_ep_, the ratio of electrophoretic velocities in FFZE and in CZE, *v*_ep,f_/*v*_ep,c_. All data are average values from three experiments with RSD values less than 2%. * *t*_r,c_ of phenol is equal to *t*_eo,c_ (the migration time of the EOF marker in CZE).

**Table 2:** Migration times, electrophoretic and electroosmotic velocities, effective mobilities and predicted and experimental distances of β-Ala-Tyr, its admixtures a_1_ and a_2_ and the noncharged component N separated by CZE and FFZE.

Sample Component	CZE				FFZE			
	*t*_r,c_ [s]	*v*_ep,c_ [mm.s^−1^]	*v*_eo,c_ [mm.s^−1^]	*m*_eff,25_ × 10^9^ [m^2^ V^−1^ s^−1^]	*v*_ep,f_ [mm.s^−1^]	*d*_eo,f_ [mm]	*d*_r,f,pred_ [mm]	*d*_r,f,exp_ [mm]
1, β-Ala-Tyr	203.5	0.864	0.561	16.7	0.0603	20	118.7	120
a_1_, admixture 1	241.7	0.639	0.561	12.7	0.0446	20	93.0	90
a_2_, admixture 2	303.2	0.396	0.561	7.6	0.0276	20	65.2	60
N, non-charged component	517.3 *	0	0.561	0	0	20	20.0	20

*t*_r,c_, the migration time in CZE; *v*_ep,c_, the electrophoretic velocity in CZE; *v*_eo,c_, the electroosmotic velocity in CZE; *m*_eff,25_, effective mobility (corrected to 25 °C); *v*_ep,f_, the electrophoretic velocity in FFZE; *d*_eo,f_, the electroosmotically migrated distance in FFZE; *d*_r,f,pred_, the predicted resulting migration distance in FFZE; *d*_r,f,exp_, experimental resulting migration distance in FFZE. All data are average values from three experiments with RSD values less than 2%. * *t*_r,c_ of non-charged component is equal to *t*_eo,c_ (migration time of the EOF marker in CZE).

## Data Availability

The data presented in this study are available on request from the corresponding author.

## Supplementary Materials

The following are available online, Figure S1: CZE separation of the standard components diglycine (Gly_2_, 0.3 mg/mL), triglycine (Gly_3_, 0.3 mg/mL) and the EOF marker phenol (N, 0.1 mg/mL). Figure S2: FFZE separation of the standard components diglycine (Gly_2_, 15 mg/mL), triglycine (Gly_3_, 15 mg/mL) and the EOF marker phenol (N, 5 mg/mL) evaluated by CZE analysis of FFZE fractions.

## Sample Availability

Samples are not available for use outside of the authors’ laboratory.
