# Supplementary material for: Application of Capillary and Free-Flow Zone Electrophoresis for Analysis and Purification of Antimicrobial β-Alanyl-Tyrosine from Hemolymph of Fleshfly Neobellieria bullata
Source: Molecules. 2021 Sep 16;26(18):5636. doi: 10.3390/molecules26185636 (PMC8469924; doi:10.3390/molecules26185636)
Supplement: Supplementary file 1 [file molecules-26-05636-s001.zip › molecules-1380530-supplementary.pdf]

molecules

MDPI

Supplementary Materials

Article

Application of Capillary and Free-Flow Zone Electrophoresis for Analysis and Purification of Antimicrobial Dipeptide β-Alanyl-Tyrosine from Hemolymph of Fleshfly *Neobellieria bullata* †

Veronika Šolínová ‡, Petra Sázelová ‡, Alice Mášová, Jiří Jiráček and Václav Kašička *

Institute of Organic Chemistry and Biochemistry of the Czech Academy of Sciences, Flemingovo n. 542/2, 166 10 Prague 6, Czech Republic; veronika.solinova@uochb.cas.cz (V.Š.); petra.sazelova@uochb.cas.cz (P.S.); alice.masova@gmail.com (A.M.); jiri.jiracek@uochb.cas.cz (J.J.)

* Correspondence: vaclav.kasicka@uochb.cas.cz; Tel.: +420-220-183-239

† Dedicated to Prof. Dr. Boguslaw Buszewski at the occasion of his 70th birthday.

‡ These authors contributed equally to this work.

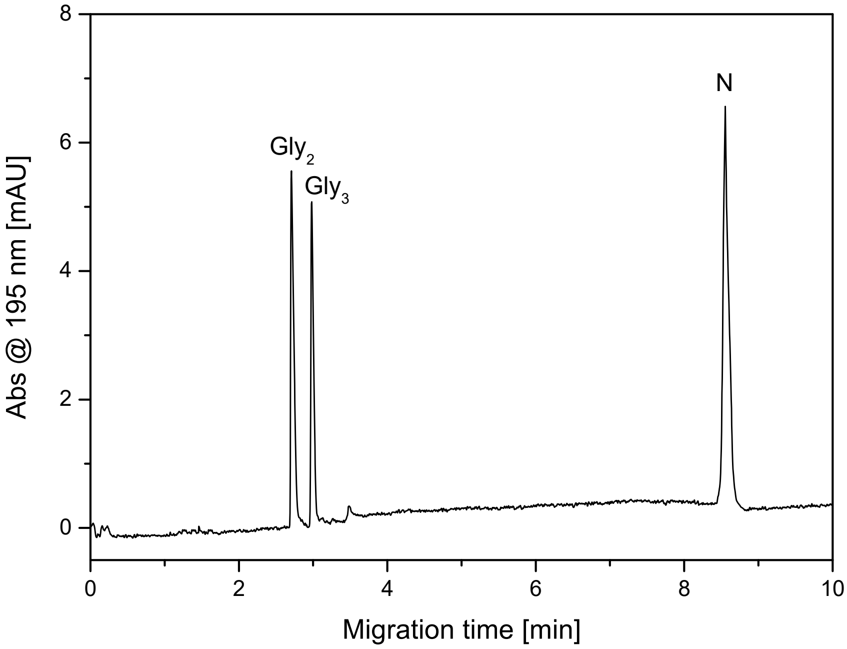

Figure S1. CZE separation of the standard components diglycine (Gly_2, 0.3 mg/mL), triglycine (Gly_3, 0.3 mg/mL) and the EOF marker phenol (N, 0.1 mg/mL). BGE: 500 mM AcOH, pH 2.50; capillary: bare fused silica, total/effective length 393/290 mm, id/od 50/375 µm; separation voltage: 20 kV; current: 11.8 µA; temperature: 20 °C. Sample concentration 0.3 mg/mL. For other experimental conditions, see Section 2.3.1.

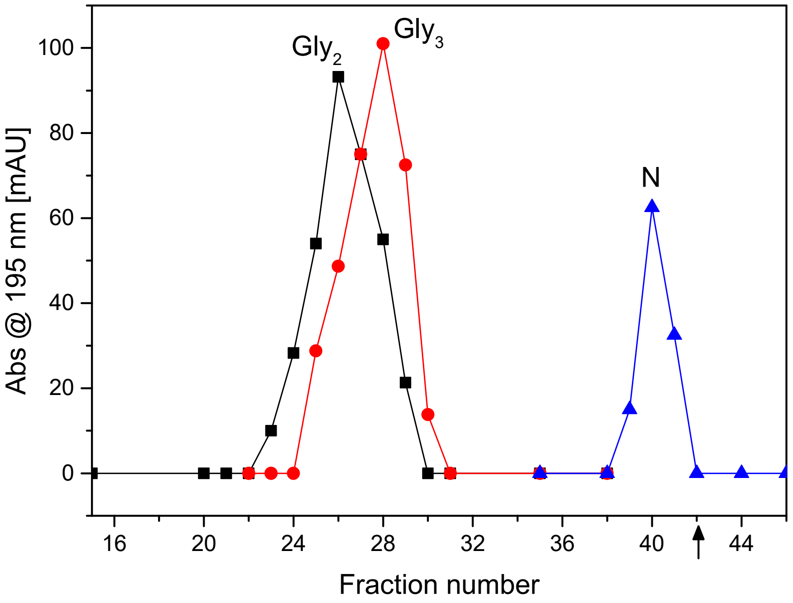

Figure S2. FFZE separation of the standard components diglycine (Gly_2, 15 mg/mL), triglycine (Gly_3, 15 mg/mL) and the EOF marker phenol (N, 5 mg/mL) evaluated by CZE analysis of FFZE fractions. Carrier BGE: 500 mM AcOH, pH 2.50; separation voltage 3.0 kV; current 122–125 mA; sample flow rate 1.6 mL/h; flow-through time 31 min. The arrow at fraction no. 42 indicates the position, at which the sample is continuously introduced at the inlet side of the flow-through (free-flow) chamber. From this position, the migration distances of the separated compounds are calculated. The length of one fraction is 10 mm. Thus, for example, in this figure, the migration distance of the neutral component, phenol (peak N) with apex of the peak in fraction 40 is 20 mm, and the migration distances of diglycine and triglycine (peaks Gly_2 and Gly_3) with the apexes of peaks in fractions 26 and 28 are 160 and 140 mm, respectively. These distances are presented in Table 1, section FFZE. For other experimental conditions of FFZE, see section 2.3.2. For experimental conditions of CZE, see Figure S1 and Section 2.3.1.
